# Double J-related hemoperitoneum in a living-related renal transplantation recipien

**Published:** 2012-09-30

**Authors:** Sh FTsai, K Shu, C H Chen

**Affiliations:** 1 Division of Nephrology, Department of Internal Medicine, Taichung Veterans General Hospital, Taichung, Taiwan; 2 Department of Life Science, Tunghai University, Taichung, Taiwan; 3 School of Medicine, Chung Shan Medical University, Taichung, Taiwan

**Keywords:** double-J, renal transplantation, hematoma, urinoma

Dear editor

Renal transplantation (RTX) is the first choice for end-stage renal disease (ESRD). Double-Jcatheter is inserted in the ureter after anastomosis of ureter to urinary bladder (UB) in most hospitals. We report a case of double-J penetration-related complication, which caused hemoperitoneum initially, and then urinomarelated sepsis after removal of Foley catheter.

The 40-year-old man with ESRD had received hemodialysis for 15 years. Living-related RTX from his wife will performed.Human leukocyte antigen (HLA) typing were A 11, 33, B 27, 44, DR 11, 14 in donor and A 11, 24, B 27, 55, DR 4, 12 in recipient. Complement-dependent lymphocytotoxicity (CDC) cross-match was negative but Luminex test was positive (A33, DR11,14). Anti-CD20 (375 mg/m2), intravenous immunoglobulin (IVIG) 2g/kg and double filtration plasmapheresis were administered before operation. After performing anastomosis of ureter and insertion of double-J catheter, very low capacity of UB with presence of pus was discovered. Graft sonography just after operation disclosed fluid accumulation over upper pole, 1cm in diameter. Urine volume (UV)/hour was decreasing: 300c.c, 200c.c., but 30 c.c. in the third hour. Fluid accumulation progressed to 2 cm in diameter from upper pole to lower pole, extending to peri-umbilicus, with taut abdominal skin and right upper limb skin. Heart rate increased to 120/min with systolic blood pressure of 140mmHg. Hemoglobin dropped from 15g/dL to 7 g/dL. Foley catheter flow was adequate but irrigation with 30 c.c. normal saline caused lower abdominal pain. Sonography-guided needle aspiration suggested hematoma:89300/cumm of red blood cells (RBC). Five hours later, right flank and upper limb ecchymosis were found. Non-contrast computed tomography (NCCT) showed perihepaitc, perigraft (especially upper pole) and subcutaneous hematoma (Figure 1B), without active bleeding. Before NCCT, plain film was taken due to abdominal fullness (1A). Two days later, the CDC cross-match was still negative, so hyperacute or accelerated acute rejection could be excluded. Daily UV was only 100 to 200 c.c. Daily sonography showed fair perfusion. Seven days later, graft biopsy showed acute tubular injury. Methylprednisolone 500mg for three days were given owing to suspected acute rejection from post-operation day 7 to day 9. UV increased gradually to over 1000 c.c. per day without diuretics. Due to very low capacity of UB after removal of Foley catheter, he needed to urinate once every hour with volume of 70-100 c.c. He was discharged 16 days after operation with serum creatinine (SCr) of 1.7mg/dL and complete reabsorption of hematoma according to follow-up NCCT (Figure 1D). Prior to discharge, prolonged drainage tube was inserted due to persistent serosanguinous fluid formation, which was confirmed as lymphocele due to presence of 2.1mg/dL of this fluid. Unfortunately, 3 days later, right lower abdominal pain and shaking chills occurred suddenly while recuperating at home. Bacteremia (Escherichia coli) with pancytopenia (WBC, 1400/cumm; hemoglobin, 7.1 g/dL; platelets, 55000/cumm) was found. His condition improved after receiving Cefoxitin and intravenous hydration. NCCT showed periurinary bladder fluid accumulation (Figure 1E). Antegrade pyelography (AP) disclosed double-J penetration and contrast leakage into the accumulated fluid (Figure 1F). The previous cause of internal bleeding was suspected to be due to the double-J penetration after review of the first NCCT (Figure 1C). The patient claimed he produced about 120 c.c. of urine every hour. After a 14-day course of intravenous antibiotics, he was discharged with SCr of 1.0mg/dL, and permanent Foley catheter.

Ureteral stents to facilitate kidney drainage was first described by Zimskind et al (1) in 1967. Even though double-J stent became a routine part of general urological practice, its prophylactic role in RTX is still controversial (2). We always use double-J stent in RTX to buttress the repair and ensure that urine flow is not impeded by inflammation and swelling. Common complications of the double-J stent include irritative voiding symptoms (80-90%) (3), urinary tract infection, hematuria, stent encrustation and stent migration. To date, there have been no reports on double-J stent penetration in grafted kidneys. The shape of the double-J catheter is designed to prevent migration of the stent’s “J”-shaped curls. Actually, its distinctive shape meant that it was clearly apparent on plain X-ray that the proximal stent loop had lost its configuration (Figure 1A)(4). Risk factors for double-J penetration-related complications were as follows. First, double-J catheters used in RTX is more rigid than in other conditions. Double-J for use in grafts is made of polyethylene and polyurethane, which is rigid and associated with urothelial erosion or fragmentation(5,6). A flexible and elastic silicon double-J is used in native kidney. Therefore, application of a double-J stent in graft increases the risk of penetration. Second, hemodialysis for 15 years reduced the patient’s UB capacity to just 70ml~100ml. This causes less space for distal curl and a higher pressure of UB, which further leads to migration of proximal coil. Third, the patient suffered from bleeding tendency due to uremia. Fourth, removal of Foley catheter increases the pressure of UB, which causes urinary leakage through the penetration of proximal curl, resulting in high risk of urinoma-related sepsis due to immunocompromised condition. Fifth, stent length depends upon the patient's height, which in our case was 163 cm, so the inserted catheter was too long. Finally, double-J insertion was retrograde, from distal ureter to pelvis, and thus there was a greater risk of penetration of renal parenchyma. In contrast, antegrade insertion, such as percutaneous nephrostomy tube, is associated with a reduced risk.

Additionally, this patient had 3 types of fluid accumulation. Just after graft operation, lower abdominal aspiration confirmed the hematoma (RBC of 89300/cumm). Lymphocele then occurred in the drainage tube because of the similar creatinine level to serum (2.1mg/dL)(7,8). Finally, double-J penetration-related urine leakage and urinoma was found by AP. Posttransplantation fluid accumulation should be clearly differentiated to measure creatinine, protein, cell count, and triglyceride (7,8). Delayed diagnosis is usually associated with a poor outcome.

In conclusion, physicians should keep in mind the possibility of double-J penetration in kidney graft patients because it may cause delayed graft function, hematoma and urinoma, especially after removal of Foley catheter for a low capacity UB. In high-risk patients, gentle insertion of the double-J stent may prevent this complication and meticulous checking of plain film is strongly recommended for early diagnosis (8).
